# Are clinicians successful in diagnosing cutaneous adnexal tumors? a retrospective, clinicopathological study

**DOI:** 10.3906/sag-2002-126

**Published:** 2020-06-23

**Authors:** Melek ASLAN KAYIRAN, Ayşe Serap KARADAĞ, Yasin KÜÇÜK, Bengü ÇOBANOĞLU ŞİMŞEK, Vefa Aslı ERDEMİR, Necmettin AKDENİZ

**Affiliations:** 1 Department of Dermatology, Göztepe Training and Research Hospital, İstanbul Medeniyet University, İstanbul Turkey; 2 Department of Pathology, Göztepe Training and Research Hospital, İstanbul Medeniyet University, İstanbul Turkey

**Keywords:** Cutaneous adnexal tumors, histopathology, sebaceous hyperplasia, pilomatrixoma, Birt-Hogg-Dubé syndrome, trichoepithelioma

## Abstract

**Background/aim:**

Cutaneous adnexal tumors (CAT) are rare tumors originating from the adnexal epithelial parts of the skin. Due to its clinical and histopathological characteristics comparable with other diseases, clinicians and pathologists experience difficulties in its diagnosis.We aimed to reveal the clinical and histopathological characteristics of the retrospectively screened cases and to compare the prediagnoses and histopathological diagnoses of clinicians.

**Materials and methods:**

The data of the last 5 years were scanned and patients with histopathological diagnosis of CAT were included in the study.

**Results:**

A total of 65 patients, including 39 female and 26 male patients aged between 8 and 88, were included in the study. The female to male ratio was 1.5, and the mean age of the patients was 46.15 ± 21.8 years. The benign tumor rate was 95.4%, whereas the malignant tumor rate was 4.6%. 38.5% of the tumors were presenting sebaceous, 35.4% of them were presenting follicular, and 18.5% of them were presenting eccrine differentiation. It was most commonly seen in the head-neck region with a rate of 66.1%. When clinical and histopathological prediagnoses were compared, prediagnoses and histopathological diagnoses were compatible in 45% of the cases. Most frequently, it was the basal cell carcinoma, epidermal cyst, and sebaceous hyperplasia identified in preliminary diagnoses.

**Conclusion:**

Cutaneous adnexal tumors are very important, as they can accompany different syndromes and may be malignant. Due to difficulties in its clinical diagnosis, histopathological examination must be performed from suspicious lesions for definitive diagnosis.

## 1. Introduction

Cutaneous adnexal tumors (CAT) are rare benign or malignant tumors originating from adnexal epithelial parts of the skin such as pilosebaceous unit, eccrine or apocrine sweat glands [1]. It is a large group of tumors histopathologically classified as follicular, sebaceous, eccrine and apocrine tumors [2]. According to the new classification made by the world health organization in 2018, Paget’s disease of the breast, extramamarian Paget’s disease, anogenital breast-like gland adenocarcinoma, hidradenoma papilliferum, and anogenital breast-like gland fibroadenoma were also included in this group under the title of region-specific tumors [3,4].

Cutaneous adnexal tumors usually present with asymptomatic papules or nodules. Although they give the clinicians a clue due to their anatomical location, number and distribution, histopathological examination is the gold standard in diagnosis [5]. Yet, due to the presence of large histomorphological patterns in the histopathological examination, it may be confusing to use many different terms together when describing the same tumor [5]. Different diagnoses may be made histopathologically outside of what is written in clinical prediagnoses due to the presenting clinical and histopathological similarities.

In this study, we aimed to compare the prediagnoses of the patients, who was diagnosed with CAT as the histopathologically confirmed diagnosis, with their histopathological diagnoses, by retrospectively scanning the files of these patients, and to examine the compatibility between the clinical and pathological results. In addition, we aimed to reveal both the clinical and the histopathological characteristics of CAT.

## 2. Materials and methods

The approval of İstanbul Medeniyet University Göztepe Training and Research Hospital Ethics Committee was obtained in order to carry out the study (2019/0484).

Patients that were admitted to our Dermatology outpatient clinic between the dates of December 2014 and July 2019 were retrospectively screened and 65 of these patients, who were histopathologically diagnosed with cutaneous adnexal tumors were included in the study.

Biopsies taken by dermatologists from patients’ lesions were placed in 10% formaldehyde solution and sent to the department of pathology. At the department of pathology, the biopsy materials were set in paraffin, prepared as thin lamina-lamella sections, and stained with hematoxylin and eosin dye. Different immunohistochemical dyes, which were necessary to diagnose some of the biopsy materials, were also administered. The biopsy preparations were examined by dermatopathologists and the related diagnoses were made. Demographic characteristics of the patients such as sex and age, and their clinical characteristics such as anatomical localization and primary lesions were recorded through scanning their clinical files.

In consequence, the data were analyzed by using software Statistical Package for Social Science (SPSS) version 16.0, and the results were expressed in percentage.

## 3. Results

A total of 65 patients, including 39 female and 26 male patients aged between 8 and 88, were included in the study. The female to male ratio was 1.5. The mean age of the patients was 46.15 ± 21.8 years. The mean age of the female patients was 44.5 ± 23.5 years, whereas the mean age of the male patients was 48.7 ± 19.03 years. Lesion times ranged from 1 month to 15 years. The average duration of lesions was 4 years. The disease was prevalent at the age range of 40–49 years at the most (18.46%), followed by the age range of 60–69 years (15.38%); on the other hand it was least prevalent at the age range of 0–9 years (0.31%), followed by the age range of 70–79 and 80–89 age (7.69%).

The benign tumor rate was 95.4%, whereas the malignant tumor rate was 4.6%. 38.5% of the tumors were presenting sebaceous, 35.4% of them were presenting follicular, 18.5% of them were presenting eccrine differentiation; 2 of them were presenting mixed differentiation, whereas another 1 was presenting apocrine differentiation, and another 2 of them were region-specific.

**Figure 1:**
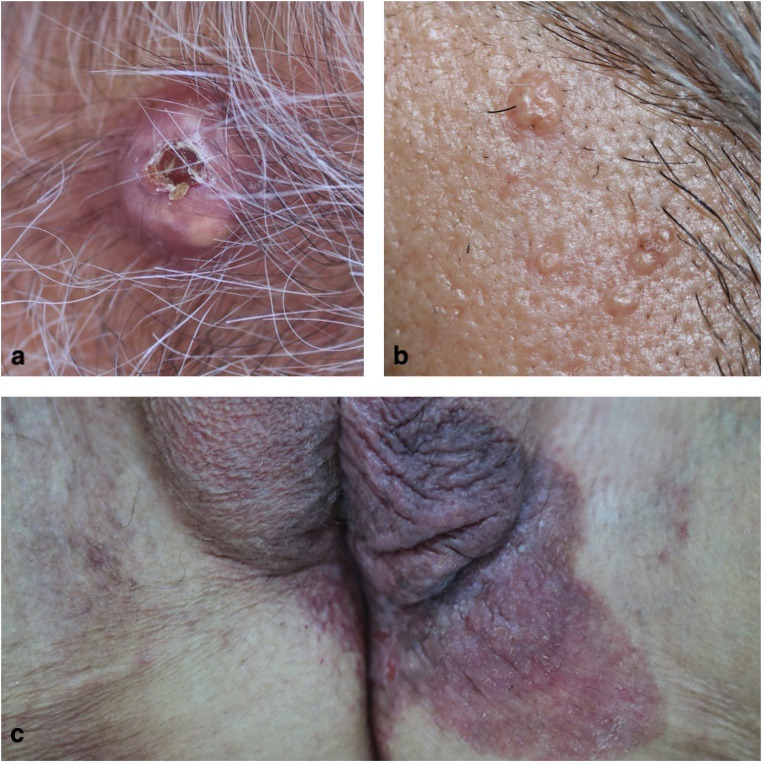
a: Pilomatrixoma-nucha region, b: Sebaceous hyperplasia-temporal region, c: Extramammary Paget’s disease-anogenital region.

The 2 most common tumor groups were sebaceous hyperplasia (35.4%) and pilomatrixomas (24.6%) (Figures 1a–1b). The most common malignant cutaneous adnexal tumor was extramammary Paget’s disease (Figure 1c). Follicular cyst, porocarcinoma, syringocystadenoma, trichoadenoma and trichodiscoma were the least common tumors, with one case each (Table 1). Three of the patients presented with complaints of neoplastic growth, whereas another 3 presented with complaints of pain, whereas 1 presented with complaint of bleeding; however most of the lesions were asymptomatic. As the primary lesion, 44.6% of them had papules, 29.2% of them had nodules, 13.9% of them had tumors, 9.2% of them had plaques, and 3.1% of them had cysts.

**Table 1:** Types and differentiations of the cutaneous adnexal tumors, sex and percentages of affected patients.

Diagnosis	Number of female patients	Number of male patients	Total percentage	Differentiation
Sebaceous hyperplasia	14	9	35.4%	Sebaceous
Pilomatrixoma	9	7	24.6%	Follicular
Trichoepithelioma	3	1	6.2%	Follicular
Eccrine spiradenoma	3	-	4.6%	Eccrine
Eccrine chondroid syringoma	-	2	3.1%	Mixed
Extragenital Paget’s disease	2	-	3.1%	Region-specific
Hidradenoma	1	1	3.1%	Eccrine
Hidroacanthoma simplex	2	-	3.1%	Eccrine
Poroma	2	-	3.1%	Eccrine
Sebaceous Adenoma	-	2	3.1%	Sebaceous
Syringoma	1	1	3.1%	Eccrine

When the anatomical localizations of the lesions were taken into consideration, 20% of the lesions were localized on the nose, 4.6% of them were localized on the eyelids and periorbital area, 1.5% of them were localized on the ears, and 32.3% of them were localized on the face. In addition, 15.4% of them were located on the hip, and on the genital or lower extremities, 10.8% of them were located on the upper extremities, 7.7% of them were located on the nucha and neck, 4.6% of them were located on the scalp, and 3.1% of them were located on the torso.

Eighteen of the cases were diagnosed by means of total excisional biopsy, 1 of the cases was diagnosed by means of shave biopsy, whereas the others were diagnosed by means of punch biopsy.

Three patients who were diagnosed with malignancy were then underwent extensive surgical operation and lymph node dissection.

**Figure 2:**
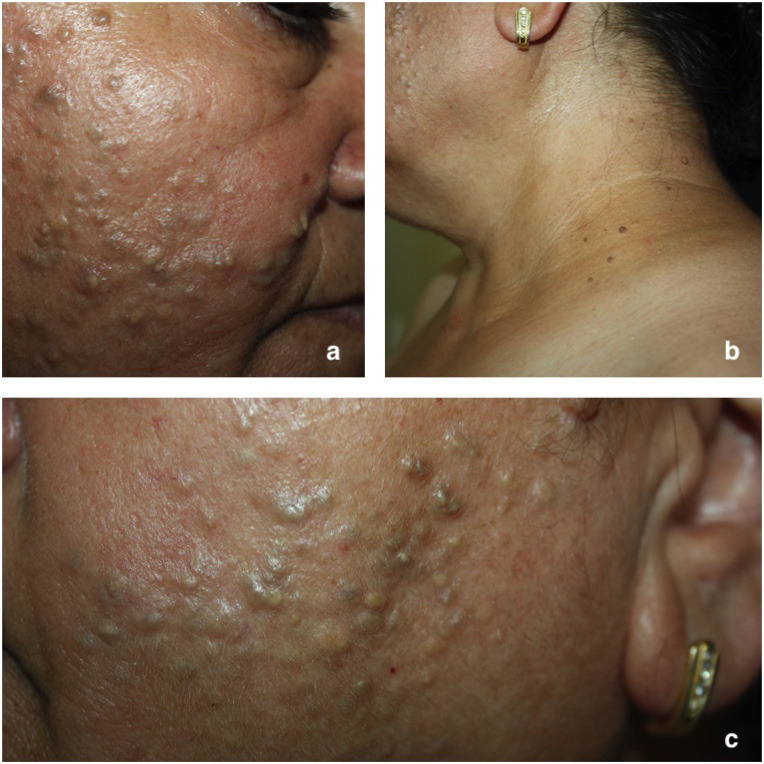
Birt-Hogg-Dubé syndrome. a and c: Follicular cysts and fibrofolliculomas are seen on the patient’s cheeks, b: Acrochordons are seen on the patient’s neck.

One of the patients was diagnosed with Birt-Hogg-Dube syndrome, another was diagnosed with Bazex-Dupre-Christol syndrome, and 2 others were diagnosed with multiple familial trichoepithelioma (Figures 2a–2c 3a and 3b). One patient was diagnosed with accompanying Koenen tumor, and another 1 was diagnosed with accompanying collagenoma.

**Figure 3:**
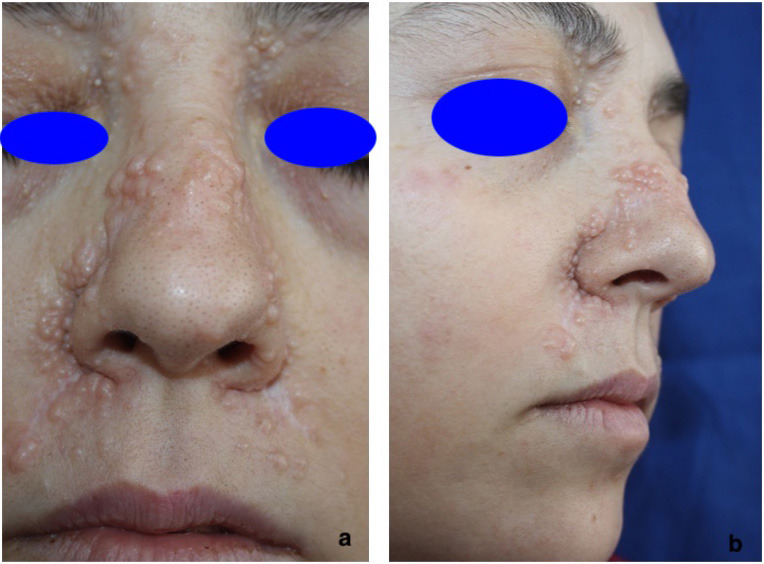
a and b: Multiple familial trichoepithelioma. Numerous trichoepitheliomas are seen on the patient’s nose and on the edges of the eye

**Figure 4:**
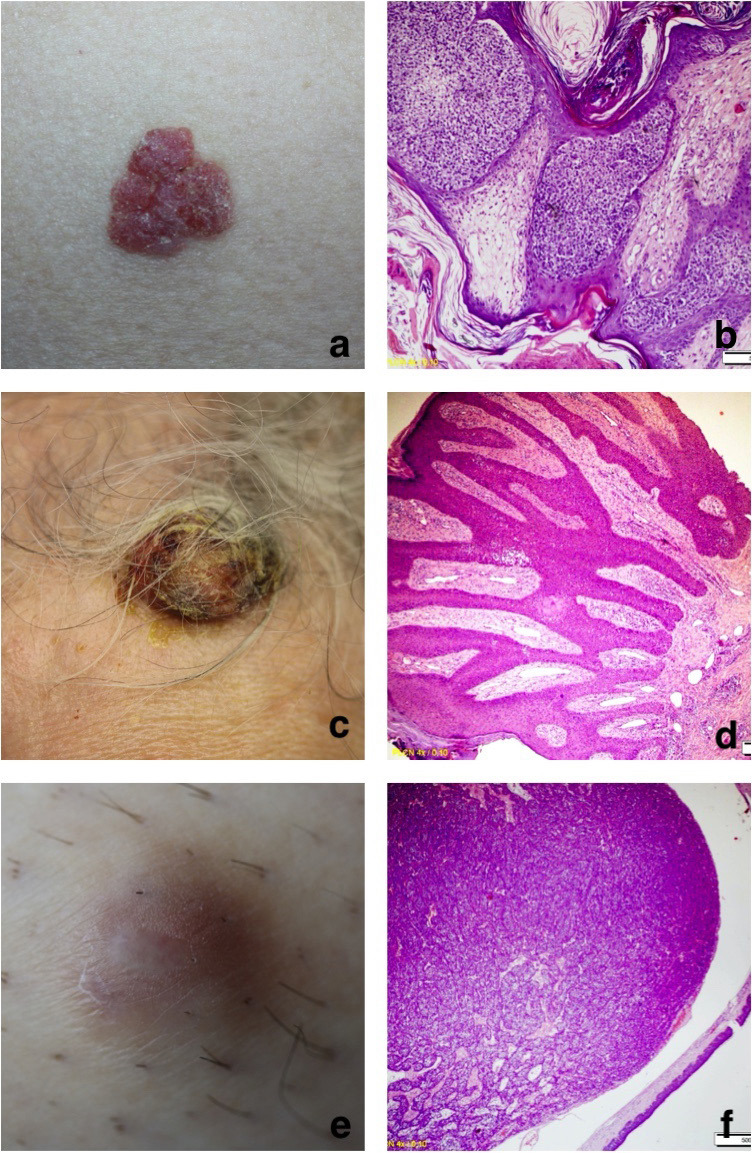
Examples of cutaneous adnexal tumors and their histopathological examination. a: Porocarcinoma, b: Histopathology of the porocarcinoma: Tumors that extend from the epidermis to the dermis, forming broad bands that anastomose with each other, are observed as islets in the dermis and have enlarged hair follicles around them. (HEX100), c: Poroma, d: Histopathology of the poroma: benign tumoral formations that are associated with the epidermis and that develop as islets that anastomose with each other into the dermis formed by relatively uniform basal cells, and as extensive funiculi. (HEX40), e: Hidradenoma, f: Histopathology of the hidradenoma: A well-confined lobulated dermal nodule consisting of cells with localized clear cytoplasm was seen under the epidermis. (HEX40).

**Figure 5:**
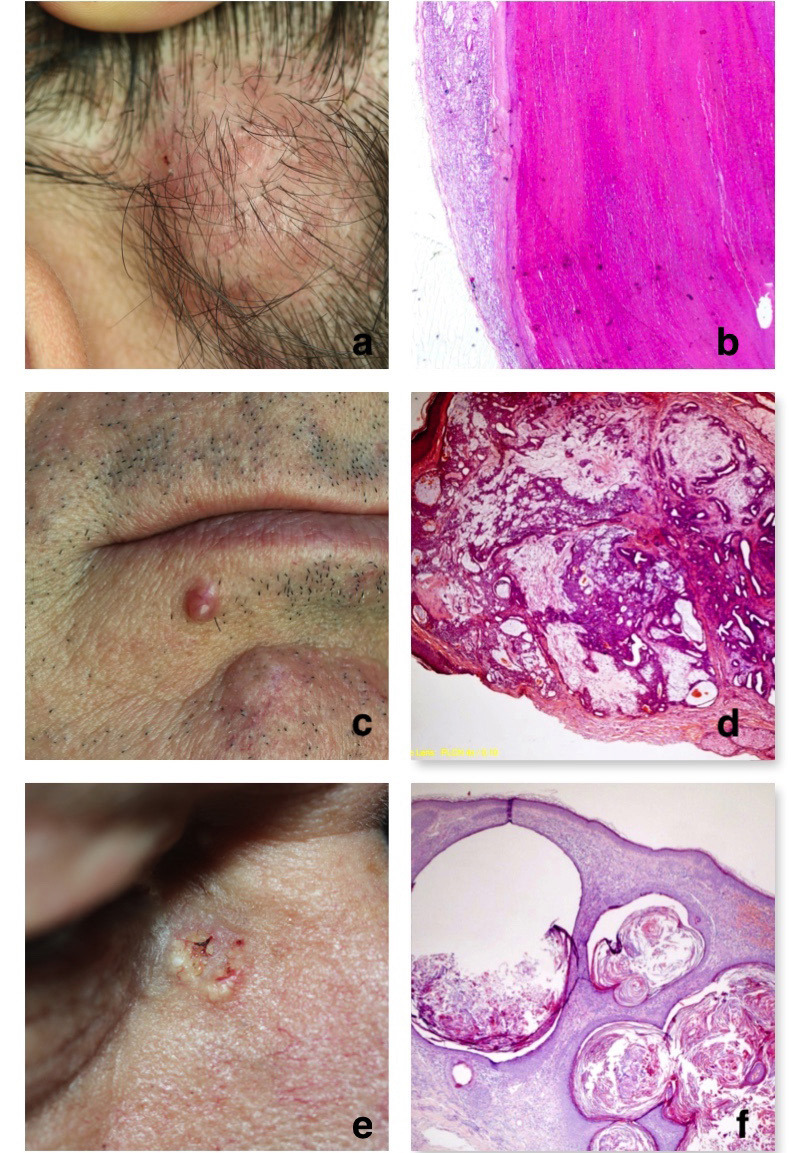
Examples of cutaneous adnexal tumors and their histopathological examination. a: Trichilemmal cyst, b: Histopathology of the trichilemmal cyst: A cystic lesion with an amorphous eosinophilic keratinized material, the surface of which is covered with squamous epithelium. (HEX40), c: Chondroid syringoma, d: Histopathology of the chondroid syringoma: Biphasic patterned tumoral tissue that combines epithelial and stromal components. The fibromyxoid stroma with localized diffuse areas of hyalinization is mostly seen. It is noteworthy that the epithelial component consists of glandular structures. (HEX40), e: Trichoadenoma, f: Histopathology of the trichoadenoma: Many oval-rounded keratinous cysts in the dermis under the epidermis in a fibrous stroma. Eosinophilic squamous epithelial cells around keratinous cysts (HEX100).

When clinical and histopathological prediagnoses were compared, prediagnoses and histopathological diagnoses were compatible in 45% of the 65 patients, whereas they were contradicting in 51% of the 65 patients (Figures 4a–4f and 5a–5f). Two of the patients were not preliminarily diagnosed. Clinical diagnoses were consistent with preliminary diagnoses in 65.2% of the cases of sebaceous hyperplasia, which was the most commonly seen type of tumor, and in 50% of the cases of pilomatrixoma, which was the second most commonly seen type of tumor.

Most frequently, it was the basal cell carcinoma (15 patients), epidermal cyst (14 patients), and sebaceous hyperplasia (14 patients) identified in preliminary diagnoses; followed by the diagnoses of pilomatrixoma (8 patients), syringoma (7 patients), sebaceous adenoma (5 patients) and verru vulgaris (4 patients), respectively.

## 4. Discussion

Across the world, benign cutaneous adnexal tumors are more common than the malignant CAT. In our study, rate of benign tumors was much more (95.4% benign, 4.6% malignant tumor rate) compared to the rates reported in the studies carried out around the world. Only the rate of malignant tumors (6.06%) reported in the retrospective study of Suri et al. was similar to the rate we have reported in our study [6]. Suri et al. have worked with 66 cases in their study, which is also comparable to the number of cases included in our study. In other studies carried out around the world, the rates of benign tumors were reported respectively as 80.36%, 77.14%, 90.48%, and 88.5%; whereas the rates of malignant tumors rate were reported as 19.64%, 29.63%, 9.52%, and 11.5%, respectively [7–10].

In our study, 38.5% of the tumors were presenting sebaceous, 35.4% of them were presenting follicular, 18.5% of them were presenting eccrine differentiation; 2 of them were presenting mixed differentiation, whereas another 1 was presenting apocrine differentiation, and another 2 of them were region-specific. It seems that sweat gland tumors were reported more frequently in other studies. For instance, in a study with 56 patients, patients had sweat gland tumors (42.86%) at the most, followed by patients having follicular tumors (35.71%), and sebaceous tumors (21.43%), respectively [7]. In another study including 33 cases, eccrine tumors (51.5%) were reported as the most frequent tumors followed by follicular tumors (36.36%). Apocrine tumors were the least common tumor type in the same with 2 cases as it was in our study. On the other hand, number of sebaceous tumor cases reported in this study was also low, with only 2 cases [11]. In a study with a larger series of 110 patients, follicular tumors (39.09%) were found to be the most common, followed by sweat gland tumors (37.27%), and sebaceous tumors (23.63%), respectively [12]. In our study, sebaceous hyperplasia (35.4%) was the most common case, followed by pilomatrixoma (24.6%) as the second most common case, whereas the most common malignant tumor case was extramammary Paget’s disease. In the literature, nodular hidradenoma, pilomatrixoma, trichoepithelioma or eccrine spiradenoma were the most commonly reported types of benign tumors, whereas the sweat gland carcinoma and sebaceous carcinoma were the most commonly reported types of malignant tumors [6,8,12–15]. The most commonly reported benign and malignant cutaneous adnexal tumor diagnoses differ from one study to the other. In a twelve-year long study, where the relevant data were scanned retrospectively and only the malignant CAT were examined, the most commonly seen malignant CAT were reported as eccrine adenocarcinoma and appendage carcinoma. It has also been reported that the prevalence rates of the said types of malignant CAT were 20% for both types, and that they were often observed in the head and neck region (52%) [16].

The female to male ratio in our study was 1.5. This rate was between 1.03 and 1.44 in most of the other studies, slightly in favor of male patients [6,7,10,12]. On the other hand, it has been reported in the Nair’s study that CAT were seen 2.3 times more in female patients than in male patients [11]. In our study, the average duration of the lesions was 4 years, which is shorter than the average duration of the lesions reported in other studies [8]. Similar to the other studies, most of the lesions in our study were asymptomatic papulonodules as well [8]. The mean age of the patients included in our study was 46.15 years, which is higher than the mean age of patients reported in other studies [10]. The most frequently diagnosed age range in our study was the age range of 40–49 years (18.46%), which was followed by the age range of 60–69 years (15.38%). The most frequently diagnosed age range varied in previously held studies. The age range of 51–60 years was reported as the most frequently diagnosed age range (26.78%) in Sharma et al.’s study, where it has been stated that CAT are commonly seen at advanced ages. On the other hand, the age range of 11–20 years was reported as the most frequently diagnosed age range (27.27%) in Nair’s study [7,11]. Similar to what we have reported, Vani et al. have also reported the age range of 40–49 years as the most frequently diagnosed age (21.56%) range in their study [17]. Some of the tumors diagnosed are actually present at early ages, however they become apparent over time as thickening and growth become more evident in adolescent age, and hence patients generally do consult a doctor after the adolescence period [6].

In our study, the region where we saw CAT most frequently was the head and neck region with a rate of 66.1%, and within this region CAT was most frequently seen on the facial region with a rate of 32.3%. The head and neck region was reported as the most frequently affected region in other studies as well [6,9]. The regions that the CAT were most frequently seen and the associated incidence percentages, which were reported in the Sharma et al.’s study, were comparable to the results reported in our study. Sharma et al. reported the head and neck region as the most frequently affected region with a rate of 64,28%, and the facial region as the most affected region within the head and neck region with a rate of %37,5 [7]. The reasonforthe cutaneous adnexal tumors to be observed most frequently in the head and neck region is related to the fact that sweat glands and pilosebaceous units are most frequently located in this area [6,18].

Two of our cases were diagnosed with multiple familial trichoepithelioma. These 2 cases were mother and son. There were numerous lesions in these cases, especially in the mother, and they were localized on the face. On the other hand, in Nair’s study, 4 of the 35 cases were reported to have trichoepithelioma [11]. Multiple familial trichoepithelioma is a disease that frequently affects the face and that has a localized autosomal dominant transition in the chromosome 9p21 [19]. In tumor histopathology, many cysts and basophilic tumor islets with palisading nucleus in the periphery are noteworthy and it may be difficult to differentiate them due to their similarity to basal cell carcinoma presenting follicular differentiation. This situation poses a challenge for the pathologist, especially when there is only a single lesion [20]. We have observed in the pathology of our patients, tumor cell islets located in the fibrous stroma consisting of basal cells and presenting follicular differentiation.

Birt-Hogg-Dubé syndrome has been defined as a syndrome presenting with lung cysts, pneumothorax, renal tumors and colon polyps, as well as CAT such as fibrofolliculoma and trichodiscoma [21,22]. However, the name of the syndrome has been renamed as Hornstein-Birt-Hogg-Dubé syndrome by some scientists since 2012, when it was noticed that the histopathologies of fibrofolliculoma and trichodiscoma were the same and hence that they were considered to be the same entity [23]. Our patient, who was diagnosed with this syndrome, was a 54-year-old female patient. She had presented with numerous yellow-gray colored smooth surface papules that are 2–4 mm in diameter in the bilateral malar region. Her dermatological examination revealed a large number of acrochordons that stood out in her neck. She was diagnosed with follicular cyst and fibrofolliculoma as a result of a biopsy taken from the malar region. The patient had stated that her sister and daughter have the same lesions.

Bazex-Dupre-Christol syndrome is a syndrome characterized by X-linked dominant follicular atrophoderma, multiple basal cell carcinoma, hypotrichosis and localized hypohidrosis [24,25]. Our 57-year-old female patient presented with yellowish-brown papular lesions that started 10 years ago as a single lesion on her face and which then spread over her entire face, and more intensely on the regions of orbit, eyebrows and nose. Biopsies taken from the lesions were reported as trichoepithelioma and basal cell carcinoma. The pathology was compatible with the basal cell carcinoma, which was presenting localized retraction artifacts, consisting of basal cells lined up in the periphery, and commonly stained with Berep-4 immune staining, and with the trichoepithelioma presenting with follicular differentiation.

It is crucial to make a diagnosis in the event of cutaneous adnexal tumors, as they may be of malignant nature and they may accompany many other syndromes. Lesions may appear as a part of many different syndromes besides being solitary. Thus it may not be possible to diagnose these syndromes before the patient actually presents with CAT. Familial cases, such as Birt-Hogg-Dubé syndrome, Cowden syndrome, Bazex-Dupre-Christol syndrome, Muir-Torre Syndrome, multiple familial trichoepithelioma, can be given as examples to such lesions (Table 2) [19,21,24,26–37]. Although very rare, CAT also have malignant forms. These tumors affect the lymph nodes and can distant metastasize. Therefore, they often have poor prognosis [38]. However, it is not always possible for clinicians to think in differential diagnosis. In our study, the compatibility rate of clinical diagnosis and histopathological diagnosis was 45%, which was quite high compared to other studies. For instance, in the study conducted by Rajalakshmi et al. on 21 patients, the clinical diagnosis of only one patient was compatible with the histopathological diagnosis, whereas in another study performed by Radhika et al. on 35 patients, clinical diagnosis of only 4 cases were found to be compatible with the histopathological diagnoses [8,10]. In a study carried out in Germany on 13 patients with pilomatrixoma around their eyes, it has been reported that none of these patients were diagnosed with pilomatrixoma during the examination, but only 3 were suspected of having pilomatrixoma, during their surgical operation [39].

**Table 2:** Table 2. Syndromes associated with cutaneous adnexal tumors [19,21,24,26–37]. (AD: Autosomal dominant; AR: Autosomal recessive; XR: X-linked recessive)

Syndrome	Heredity and mutation	Observed cutaneous adnexal tumors	Systemic findings	Important notes	Affected regions
Brooke-Spiegler syndrome	ADCYLD gene mutation(80%–85%)	*Spiradenoma *Cylindroma *Spiradenocylindroma *Trichoepithelioma	*Rarely salivary gland involvement. *In 5%–10% of patients, malignancy develops from existing cutaneous adnexal tumors.	Multiple familial trichoepithelioma is a phenotypic variant of this disease that is only available with numerous trichoepitheliomas on the skin.	*The middle region of the face *Rarely scalp, neck, back
Cowden syndrome	ADPTEN gene mutation	Trichilemmoma	*Thyroid gland abnormalities *Often bilateral breast cancer and other breast diseases *Ovarian cyst, renal and endometrium cancer *Endometrial and gastrointestinal polyps	*Keratoses on the distal extremities. *Benign fibroma and papillomas in the oral mucosa (cobble stone appearance) *Palmoplantar hyperkeratosis multiple skin tag	Face, especially nose, nasolabial sulcus and cheeks
Muir-Torre syndrome	ADDNA mismatch repair gene mutation	*Sebaceous tumors *Keratoacanthoma	*Visceral malignancy (colorectal, genitourinary, breast, hepatobiliary)	With the exception of sebaceous hyperplasia, Muir-Torre syndrome should be excluded in every patient diagnosed with multiple sebaceous tumors.	Face and neck
Rombo syndrome	ADSilent mutation	Trichoepitheliomata	Peripheral cyanosis	*Basal cell carcinoma *Atrophoderma vermiculatum *Milia *Loss of eyelashes and eyebrows *Hypotrichosis, and telangiectasia may occur.	*Cheeks, preauricular area and forehead, *Atrophoderma is seen on thecheeks and elbows.
Gardner’s syndrome	ADAPC gene mutation	*Pilomatrixoma *Epidermoid cysts *Trichilemmal cysts *Hybrid cysts	*Gastrointestinal polyps and adenocarcinoma, *Papillary Thyroid carcinoma, *Mesentery or bone desmoid tumor, *Osteosarcoma, *Chondrosarcoma, *Hepatoblastoma, *Mandibula and skull osteomas, fibromas, *Brain tumors *Congenital hypertrophy of the retinal pigment epithelium	Gardner’s fibroma is often seen in the torso and its histopathology consists of thick, randomly arranged collagen bundles and scattered fibroblasts.	Gardner’s syndrome should be excluded in the presence of tumors, especially if exists in large numbers in the face, scalp, and extremities.
Birt-Hogg-Dubé syndrome	ADBHD/FLCN mutation	*Fibrofolliculoma *Trichodiscoma *Perifollicular fibroma	*Renal carcinoma *Spontaneousrecurrentpneumothorax *Medullary thyroid carcinoma, multinodular goiter, thyroidadenoma	Acrochordon, angiolipoma, lipoma, angiofibroma, angiomatous nodules	Face, neck and upper torso
Bazex-Dupré-Christol syndrome	XDMutation unknown	*Trichoepithelioma, *Follicular Atrophoderma	*Congenital hypotrichosis, *Atopic tendency	*Multiple Basal Cell Carcinoma *Multiple milia on the face *Hyperpigmentation on the forehead	For atrophoderma; dorsal part of the hands and feet and over the knees-elbows
Schöpf–Schulz–Passarge syndrome	ARWNT10A gene mutation	Apocrine hidrocystoma	Hypodontia	*Palmoplantar keratoderma *Hypotrichosis, *Nail dystrophy	Periocular region andeyelids

While diagnosing cutaneous adnexal tumors, not only the clinicians, but also experienced dermatopathologists may find it difficult to make a diagnosis and determine the type of the tumor [40]. 

In their study involving 68 cases, Tirumalae et al. aimed to reveal histological features on silhouettes that would facilitate the separation of malignant and benign CAT [41]. Ackerman made this distinction for the first time for melanoma and Spitz nevi, which are 2 diseases in the field of dermatology that are difficult to distinguish, and the said method of Ackerman was also used by Tirumalae et al. [41,42].

Cutaneous adnexal tumors are quite important as they can be confused with malignant tumors, accompany different syndromes, and they may be malignant themselves. However, due to the similarity of the clinical and histopathological features they demonstrate, clinicians and pathologists may have difficulty in making a diagnosis. For a definitive diagnosis, histopathological examination must be performed from suspicious lesions, and CAT should also be included in the differential diagnosis of lesions, particularly the papulonodular lesions, on the skin that do not demonstrate any specific features.

## Disclaimers/Conflict of interest

There are no financial conflicts of interest to disclose. There are no conflicts of interest among all authors regarding this article. 

All authors have contributed significantly, and all authors are in agreement with the content of the manuscript. Our study has been presented as an oral presentation in 24th Prof. Dr. Lütfü Tat Symposium held in Ankara between dates November 20–24, 2019.
